# In time: Eosinophilic esophagitis: when to suspect it and how to diagnose it in children and adolescents

**DOI:** 10.1016/j.rppede.2016.07.001

**Published:** 2016

**Authors:** Mirna Chehade

**Affiliations:** Mount Sinai Center for Eosinophilic Disorders, Jaffe Food Allergy Institute, Icahn School of Medicine at Mount Sinai, New York, USA

## Prevalence and demographics

Eosinophilic esophagitis (EoE) is a chronic immune, antigen-mediated, disease of the esophagus characterized by symptoms related to esophageal dysfunction and significant esophageal eosinophilic infiltration.^1^ EoE has been described in many places throughout the World, including North America, Europe, South America, Australia, Asia, and the Middle East. There are no reported EoE cohorts in sub-Saharan Africa or India.^2^ Multiple reports have originated in Brazil, including São Paulo, of children with EoE.^3^^-^ 5 The prevalence of EoE has been steadily increasing,^2^ therefore, it is important for pediatricians and pediatric specialists of various disciplines to be familiar with the disease presentation, so that diagnosis can be made in a timely manner, and optimal care can be provided.

EoE is more common in boys, with a male:female ratio of 3:1, and can present at any age in children, including in infancy.^6^ Familial clustering has been reported in EoE,^7^ and was found to be due in a larger part to shared family environment than to genetics, the latter being caused by a complex rather than Mendelian inheritance.^8^ A number of early life exposures such as antibiotic use in infancy, cesarean delivery, preterm birth, and formula-only or mixed (infant formula and breast milk) feeding were thought to be potentially associated with the development of EoE in the pediatric population.^9^

50-70% of children with EoE have concomitant atopic diseases, including asthma, allergic rhinoconjunctivitis, or atopic dermatitis. In addition, a large number of children with EoE have current or past history of food allergy.^1^ Family history of atopy is present in a large number of children with EoE.^10^

## Clinical presentation

Children with EoE present with a variety of symptoms, depending on their age and the duration of their disease. Symptoms include abdominal pain, gastroesophageal reflux (GER) symptoms including nausea and emesis, solid food dysphagia, and esophageal food impactions.^10^

A few challenges can face the clinician in this area. The first one is that children with EoE present at times with infrequent or non-specific symptoms, therefore not perceived as alarming to the families or the clinician. While adolescents and older children mostly report dysphagia and food impactions, younger children and patients with shorter duration of symptoms are more likely to present with abdominal pain, GER symptoms, and occasional emesis.^11^ Discerning EoE from acid-induced GER disease in these patients by history alone can be difficult. Inquiring for other associated symptoms such as early satiety, and assessing for the presence of failure to thrive can be very helpful, as these points to the possibility of EoE. In fact, failure to thrive can occur in up to a third of children with EoE,^10^ and is potentially reversible following disease remission.

A second challenge faced by the clinician is that symptoms can be subtle in nature, given that the disease is chronic and its symptoms evolve over time. Therefore, children with EoE learn to compensate through behavioral modifications in feeding patterns to prevent major symptoms such as emesis, dysphagia or esophageal food impactions. These behaviors include avoidance of large meals, avoidance of foods that have hard or lumpy textures such as meats and breads, prolonged chewing, cutting food into smaller pieces, lubricating food bites with condiments, and drinking with most bites of food.^1^ This emphasizes the importance of obtaining a detailed history from both children and adolescents with suspected EoE and their families to prevent a delay in diagnosis.

## Diagnosis

The diagnosis of EoE requires performing an upper endoscopy with multiple biopsies of the esophageal mucosa as well as other parts of the gastrointestinal tract. Visual inspection of the esophageal mucosa can reveal one or more findings,^12^ including furrows, white plaques and loss of vascular pattern, all common in the pediatric population. While the cause of furrows is unclear, white plaques are formed by aggregates of eosinophils closest to the luminal surface associated with some sloughing of the superficial epithelial cells.^13^ In addition, esophageal rings, strictures, narrowing, or even shearing can be present in more severe cases. A combination of features is often present. In up to 20% of children with EoE, the esophagus may appear completely normal, highlighting the importance of obtaining biopsies at all times whenever EoE is clinically suspected.^14^

Since EoE is a patchy disease, multiple esophageal biopsies are needed from various locations of the esophageal mucosa, especially from lesional areas such as white plaques. Esophageal biopsies demonstrating at least 15 eosinophils per high power field in the most densely infiltrated area upon microscopic examination of hematoxylin and eosin-stained sections are considered diagnostic, in the absence of increased eosinophilia in the remainder of the gastrointestinal tract.^1^

Since acid-induced GER disease can also result in esophageal eosinophilic infiltration, though mild, this possibility needs to be ruled out. In addition, the entity of proton pump inhibitor-responsive esophageal eosinophilia, currently considered a separate entity until its pathogenesis is elucidated, needs to be ruled out before establishing the diagnosis of EoE. Therefore, an empiric therapy with a proton pump inhibitor at a dose of 2mg/kg/day in children, up to a maximum of 20-40mg once or twice daily in adolescents, is recommended. Esophageal biopsies demonstrating significant esophageal eosinophilia despite at least 8-12weeks of this therapy are considered diagnostic for EoE.^1^

## Conclusion

In conclusion, EoE is an increasingly prevalent disease in the pediatric population. Since symptoms can be subtle, non-specific or infrequent, obtaining a thorough history focusing on a large number of symptoms including feeding history and patterns, recording personal and family history of atopy and EoE, and assessing growth are important. These can cue the pediatrician to the disease, and allow timely referral for further work-up and management.
